# Some Remarks on the Origin and Progress of the Malignant Yellow Fever, as It Appeared in the Village of Catskill, State of New York, during the Summer and Autumn of 1803, in a Letter from Dr. Benjamin W. Dwight to Dr. Eneas Monson, of New-Haven, Connecticut

**Published:** 1805-04-01

**Authors:** 


### Some Remarks on the Origin and Progress of the malignant Yellow Fever, *as it appeared in the Village of Catskill, State of New York, during the Summer and Autumn of* 1803, *in a Letter from* Dr. Benjamin W. Dwight *to* Dr. Eneas Monson, *of New-Haven, Connecticut.*

( From the Medical Repository of New-York. )

As much pains have been taken, in various newspaper publications, to persuade the people of this country that the yellow fever is never of domestic origin, but an imported disease, I have been induced to state to you some facts which appear to me to support the contrary opinion. It was originally my intention to state, generally, the existence and progress of the disease in this place, during the summer and autumn of the present year, and the manner in which I conceived it to originate; but as I have been informed that some gentlemen, who discover the nature of yellow fever by intuition, and not by observation and reasoning, have doubted the existence of the disease in this place, or have determined, if it did exist, that it must have been imported, I have thought it advisable to state *particularly* some of the first cases which occurred, that every one may judge for himself whether it was yellow fever or not.

The attending physicians were of one opinion as to the nature and origin of the disease. If they were mistaken in their opinions, then the following observations will avail nothing as to the point in debate. If their opinions were correct, then I flatter myself that it will unanswerably appear, from the following observations, that the malignant yellow fever may be, and has been generated here. That its origin is always *domestic*, I am far from believing. That it is sometimes *contagious*, there appears to be good evidence. An instance or two of its contagious nature I could produce. That it was contagious in any instance here, during the present season, there are no good grounds for believing.

The question is very important to be settled. If the disease be imported, the strictest quarantine laws should be made, that this fiend may be prevented from again landing on our shores, and brandishing the sword of destruction over our heads. If it be generated among ourselves, it is a duty incumbent on the legislature to compel people every where to remove all filth, to root out, so far as is practicable, practicable, all the causes of vegetable and animal putrefaction, and to cherish cleanliness as a cardinal virtue.

If ever the question is decided, it must be by facts. One important fact is of more avail, and will have more weight with candid men, than all the hypotheses even of the learned Haygarth, or any other the most ingenious physician or philosopher that ever lived. To facts then be the appeal.

Previously to entering upon the subject, I shall make some observations upon the local situation and circumstances of Catskill, and also upon the weather of the summer and autumn. I regret that these will not be more complete. Having resided in the place but a few months, I have not been able to possess myself of all the facts which it might be interesting to know. For a number of them, it is with pleasure that I acknowledge myself indebted to my worthy and ingenious friend, Dr. Croswell.

The village of Catskill, or, as it is usually called in the neighbourhood, Catskill Landing, is situated along the Catskill Creek. This creek empties into the Hudson about six miles below the city of Hudson, and one hundred and twenty-five above New York. Catskill Creek is formed by the junction of the Kaaters'-Kill and the Kaat's-Kill,* two streams of moderate size; the former of which, and not the latter, as is erroneously stated in the Medical Repository, Hex. I. vol. i. p. 310, arises from a pond or lake in the Blue Mountain, and, pursuing a serpentine course, empties its waters into the Kaats' Kill, about three miles from the mouth of Catskill Creek. The latter, if I am correctly informed, arises from a swamp called by the Dutch Eckeson Fly, in the town of Middleburgh, in Scoharie county, and proceeding also in a serpentine direction through Bristol, Rensellaerville, Freehold, Canton, and a part of Catskill, unites with the Kaaters' Kill. The Kaats'-Kill is the largest stream. Both have rapid currents, and in many places rocky bottoms. In both are a number of falls of considerable size and importance; some of which are in no small degree interesting and remarkable.

The creek has a bar of sand at the mouth, over which in common tides, there is about nine feet of water. This bar is the commencement of a small island, situated in the Hudson, a little north-east of the mouth of the creek. Were a wharf erected, extending from the main land, i.e. from

* This is often written Catskill.

from Catskill Point, to and along this island, any ship which could pass the mouth of the Hudson might come to Catskill with entire ease and safety, and be hauled along side the wharfs. Whenever this is done, the growth of Catskill will probably be rapid, as few towns on the Hudson are so advantageously situated for commanding the trade of a great portion of the western country.

The creek, tracing it from its mouth, proceeds in a north-western direction about three-fourths of a mile. It then takes a turn nearly west, and proceeds in this direction thirty or forty rods. After this it takes a second turn nearly north, and proceeds in that direction about three-fourths of a mile. To trace it further is of no consequence to the present design. It is navigable for vessels drawing twelve feet of water about a mile from its mouth, if they can be got over the above-mentioned bar. About a mile from its mouth there is another bar, but sloops of 100 tons may easily pass sixty or eighty rods further. The tide rises, at common tides, from four to six feet, as far up as Van Vechten's mills, about two and a half or three miles from its mouth. About one and a quarter or half a mile from its mouth there are rocks and small rapids which obstruct navigation.

The creek is of different widths in different places; it is generally from twenty to forty rods wide from its mouth to that place where the rocks and rapids commence. After this it is narrower. The current is brisk. There are no low swampy places along its banks, where the water stagnates; the banks being generally abrupt, and considerably higher than the surface of the water.

The water of the creek is soft, and makes a good lather with soap, and is generally used for washing by the inhabitants.

Including dwelling houses of all descriptions, there are more than a hundred in the village, and also a considerable number of stores. These, excepting about six or eight, are situated on the east side of the creek, and principally along one street. This street commences at the bank of the Hudson, and runs about a hundred rods in a north-west direction, near to the creek. Where the creek takes the first turn, as above-mentioned, the street is changed from a north-west course to one near north, and continues in this direction about half a mile. This part of the street is, *generally*, from ten to thirty rods distant from the creek; in some places the distance may be forty rods. After this, the road takes a second turn nearly west, till it reaches reaches the bank of the creek, and then pursuing a general north-western *direction*, is continued on the great western turnpike, leading to the Susquehannah river. Where the creek makes the first bend, there is also a short street running nearly parallel to it. On this street stand several houses, and between the houses and the creek a few stores. The ground on which these stores stand, is partly or wholly made ground. At this bend of the creek, a kind of bason is formed, and several families live, as it were, on its brim; or, in other words, some of the houses are situated upon the edge of the bank, or directly down it, one end being at the top, the other end at the bottom. Let this place be remembered, for here the sickness commenced, and around here it made all its ravages. There are some other cross lanes where houses and stores are situated. A number stand near the creek, on no regular street, and a few scattering ones are to be found elsewhere.

That space, or portion of ground, between the east side of the main street and the creek is low, and nearly level, but there is a gradual descent sufficient for conducting off all water to the creek. The eastern side of the street, throughout its whole extent, is bounded by the foot of a hill of some height. This hill has a pretty steep ascent; its height is generally, I should suppose, from fifty to a hundred feet. The northern part of the village is likewise bounded by hills of about the same height. On the western side of the creek, for the space of nearly a mile, the hills are high, and approach close to its edge, there being little or no flat. Above this, the ascent is small. The present settlement is almost entirely hemmed in by hills. The circulation of the air in the summer and autumn is, of course, often languid, and the heat and sultriness of the weather is unpleasant and relaxing.

The soil, both on the hills and in the plains, is, generally, a very fine clay. There are some small patches of loam and sand chiefly on the hills. When a rain falls, the water, instead of settling in the ground, descends in a considerable measure into the flat, and from thence into the creek, or what is not unfrequently the case, settles in the gutters and sunken places in the streets and elsewhere. Here it continues in hot weather till it is evaporated by the heat of the sun. In the course of a few days it becomes covered with a green scum, which is putrid and highly offensive, so as not unfrequently to excite disagreeable sensations in the head and stomach. In long continued hot weather, preceded by considerable rains, and aided by other local causes, some severe cases of sickness have, in several instances, been in this way produced. All this might have been prevented by a little pains-taking. By making a sufficient number of sluices, and keeping them free from obstructions, the water might easily be conducted into the creek, and in its course be very advantageously made to cleanse all sewers and drains for conducting off filth.

The well-water in Catskill, and generally in the neighbouring country, is very indifferent. Most of it, as far as my observation has extended, is hard, and abounds in hot weather with animalcules, in shape resembling tad-poles, and not unfrequently with small worms. After standing a little time, it deposits a clayey sediment. By drawing out the water freely, it may be much improved in softness and purity. Possibly all these evils might be remedied, by placing a layer of sand on the outside of the stones, a foot or two in thickness, and extending from the bottom to the top of the well; and another layer at the bottom of the well. This I suggest merely for experiment. I know not that the trial was ever made.

A free use of the water, as drink, is very apt to induce troublesome diarrhœa, especially with such persons as have resided here but a short time.

The people of Catskill live very much crowded together, most of the houses containing two, three, four, or more families.

The number of inhabitants in the village, or in the town of Catskill, which includes *the landing*, Loonenburgh, and several small settlements, cannot be ascertained. The late census, it is said, was very inaccurately taken.

This place is usually as healthy as most other places situated along the Hudson. Albany, Loonenburgh, and Hudson are probably quite as unhealthy, if not more so. Some cases of bilious remitting fever occur almost every season, but they are not usually more frequent, nor attended with more severe symptoms, than the same disease is elsewhere. In a few instances there have been cases of malignant fever, but never any considerable number previous to this season. In most instances they have clearly been owing to local causes, and have generally been confined to those places where such causes operated in an especial manner. Till the present year, the greatest number of cases have occurred in places considerably remote from the wharfs. The reason is obvious. In addition to the stagnant waters in the gutters, &c. as above-mentioned, not more than two years ago a slaughter-house was opened near near the middle of the main street, a little east from the road. Here a large number of sheep and neat cattle were slaughtered during the summer season. All the offals were thrown into the yard, and lay there from season to season. After every rain, the water which proceeded from this yard ran into the street, and there became either partially or wholly stagnant. In the neighbourhood of this sink of filth and poison it has, in several instances, been very sickly. Some cases highly malignant have, at times, occurred. In one small house, in particular, so situated as to take more of the stench than any other, Dr. Croswell informs me, that the number of cases of severe sickness, during the existence of this evil, among the several families who have resided in it, have been very remarkable. He could generally determine before-hand, from the weather, and the state of the slaughter yard, and the sewer leading from it, whether these families were to be visited with sickness or not. Some other causes of a similar nature have, at times, existed in other parts of the village, and produced sickness. In those places free from such nuisances, it has, in general, been very healthy. During the present season these evils have ceased to operate. The weather also, from being very dry since about the middle of August, has been unfavourable to the putrefaction of animal and vegetable matter. The consequence has been, that every part of the village, except the one above-mentioned, has been very healthy. Could the town of Catskill obtain an act of incorporation, with an energetic police, it would be of no small benefit.

Had the inhabitants, instead of seating themselves on the flat land by the creek, erected their dwelling houses on the hills, surrounding them in front with clean and spacious court-yards, ornamented with trees, and, in the rear, with good gardens; they would have enjoyed, in addition to a very romantic prospect, an atmosphere seldom exceeded in coolness and purity, and would have lived much more pleasantly in a variety of respects.

Building on the hills has been lately commenced; and the example being set, it will probably be followed by many others.

A predisposing cause, of no inconsiderable power to the diseases of autumn, may be found in the diet of a considerable portion of the inhabitants. Not a small number eat much too freely of *fresh meats;* the market for vegetables is, at times, badly supplied, and many families are in a great measure destitute of gardens; hence they do not use a sufficient proportion of vegetable aliment. Very little cyder or good beer is made in this part of the country, of course ardent spirits are used much too freely. The markets, however, in these and other respects, are rapidly improving, and the probability is, that in a short time the supply will be abundant.

### Weather.

I proceed now to make a few observations upon the weather. These will necessarily be incomplete. No thermometrical observations are made in the town within my knowledge. Previous to the month of July, a day or two before the commencement of which I arrived here, the state of the weather is unknown to me. Some of the observations which I noted down from time to time since then here, follow.

During the first week in July, the weather was warm, the sky clear, the ground dry. From this time to the 22d, it was, with little exception, intensely hot. I have no recollection of any such long-continued period of extreme heat. The sky was clear, little or no rain fell during the whole time, of course the ground became much parched. The crop of hay in the country around was greatly cut short, in consequence of the drought. From the 22d the weather became cooler, and continued so for a few days. A considerable quantity of rain fell on the 24th. From this time to the 30th it rained every day, and several days largely, so that the earth was soaked with water. During this period also, there was considerable lightning and thunder.

August. From the 1st to the 18th a great quantity of rain fell. During this period it rained oftener than every other day. It descended not in what are called settled rains, but in sudden, and, in some instances, heavy showers, of which there would frequently be several in the course of the day. After the sky became clear, the weather was hot and sultry, the rains not serving to cool and purify the atmosphere at all. The common remark of the inhabitants was, that there was no elasticity in the air. Though I have said that the weather was hot, yet, during the latter part of the time, there were several very cool nights, succeeded by damp, chilly, foggy mornings, the middle of the day only being very hot. From the 18th to the 25th the evenings and mornings were very cool, and generally very damp, and in several instances there was a thick fog. The 20th there was a white frost on on some low grounds in the village. The sky, during this period, was generally clear, but several days it was partially overcast with clouds. From this time to the 30th, it was clear and very hot during the day; the evenings were damp and chilly; no rain has fallen since the 18th. From the 30th of August to the 12th of September the weather was cool, but the difference between the heat of mid-day and the coolness of the evening was great; so that a change of clothes was very pleasant. From the 13th to the 20th the weather was warm in the middle of the day, and cool in the morning and evening. The 18th and 19th there were white frosts. From this time to the 25th it was warm—the remainder of the month was cool. All the rain that fell from the 18th of August to the 26th of September did not wet the earth two inches. The 26th a moderate rain.

In the month of February, Dr. Croswell informs me, the scarlatina anginosa began to prevail. It gradually increased till the month of March, when it was uncommonly rife; after this it generally decreased till it disappeared in May. A great number of children, and a considerable number of adults had the disease. It was not malignant, only one person having died of it. The local affection in the throat was, in some instances, however, considerably severe.

In May and June, intermitting fever was uncommonly frequent. More instances of it occurred at this time than during the three preceding years. It yielded very readily to the bark.

In the month of July, a considerable number of cases of cholera infantum occurred, and among adults a few cases of dysentery; but these diseases were neither more prevalent nor malignant than they generally are in this place at the same season of the year.

In the month of August the epidemic, which is the subject of the following remarks, commenced its career. The two first cases occurred on the 10th; the third and fourth on the 11th; the fifth and sixth on the 19th. The four persons who first had the disease, were seized within twenty-four hours of each other. The three who were first seized lived in one house, and they composed the whole of the family at that time.

The first person attacked was Mrs. H. This case I shall relate with a degree of minuteness that may seem to some persons tedious and uninteresting. This I shall do, be-

 cause cause the case appears to me interesting in itself, and because it is of importance to establish the nature of the disease.

CASE. I.

Mrs. H. about forty years of age, has a large frame, but is of a delicate and irritable habit. She has been heretofore much troubled with flatulency and other symptoms of dyspepsia. She began to complain, Wednesday, August 10th, of general indisposition, such as languor, lassitude, want of appetite, and other symptoms, such as usually precede fevers. Not long after, a chilly fit came on. The symptoms soon becoming more urgent, Dr. Croswell was sent for. He saw her about eleven o'clock A. M. Her pulse was very full, quick, and hard for her habit; her face was considerably swollen and much flushed; her eyes of a fiery red, the vessels being turgid with blood; her tongue was moist and covered with a whitish fur; there was a stinging heat in the skin. She complained of great pain in the fore part of the head, and in the eyes, and about the loins; she was exceedingly restless and uneasy, anxious and desponding; her abdomen was swollen and tense; her distress at the stomach was very severe; she eructated much gas, and constantly called for cold water. He ordered an injection, and administered a dose of calomel.

At five o'clock P. M. Dr. C. having ridden out of town, I was sent for. All the above-mentioned symptoms prevailed to a great degree. She said that she was in no respect easier than in the morning, but rather worse. The injection had operated once or twice, the medicine had not. What medicine had been given, or what purpose it was intended to answer, I could not learn. From the swollen face, the excessive uneasiness at the stomach, and the frequent retching, I was inclined to think that some poisonous substance had been taken into the stomach, and ventured, notwithstanding the distension of the abdomen, &c. to prescribe an emetic of *tart. emet.* and *ipecac.* At this time came in Dr. C. and approving of what I had done, the emetic was accordingly administered. It did not operate well, though it was apparently of no disservice. The calomel which she had taken in the morning soon after operated freely, but her distress and uneasiness were not at all relieved. Not long after Dr. C. administered a small opiate, and made free use of the aq. ammon. acet. made with a considerable prevalence of the alkali kali. Thursday, 11th, he commenced the use of calomel in small and frequent doses, and continued the aq. ammon. acet. Friday, 12th, at three P. M. I visited her in consultation. She was still very restless and uneasy, and complained of excessive burning and extreme sickness at the stomach; made frequent efforts to vomit, and occasionally puked a little greenish matter, which was exceedingly acrid. The inflammatory symptoms were, however, evidently abating. We continued the calomel, and instead of the aq. ammon. acet. commenced a very liberal use of sal. tart. This had an excellent effect. It relieved, to a very considerable degree, the extreme sickness and burning pain in the stomach, which no other medicine appeared to affect at all.

Her drinks were toast water, rice water, and chicken water; in the last of which a strong solution of sal. tart. was administered frequently, and in liberal quantities. The chicken water sat easter upon the stomach than any other food.

In forty-eight hours from the time of seizure, the fur flaked off from the tongue, and left it very red and dry.

Saturday, 13th. She appeared considerably better; her pulse was nearly natural; her countenance had become considerably clear; she was much less restless and desponding; her stomach was less irritable; she conversed rationally. At this time considerable yellownesss appeared in her eyes, and a less degree of it over her whole face. We ordered the medicines which were prescribed yesterday to be continued.

Sunday 14th. In the morning she appeared to be still mending. She was nearly or quite free from fever. In the course of the afternoon she became very anxious for her husband, who was severely sick with the same disease. Monday morning her anxiety, on this score, greatly increased. His death, which happened in the afternoon, agitated her exceedingly. At two o'clock P. M. the countenance had assumed a dingy appearance to a considerable degree. This afternoon she vomited a considerable quantity of black matter resembling coffee grounds. Soon after her vomiting ceased, a violent *hæmorrhage uteri* came on. It was at first very offensive, and in clots. It afterwards became of a more healthy appearance.

Tuesday, 16th. She was extremely feeble and languid; her pulse was frequent and thread-like. Her drinks were chicken broth and porter, wine and brandy, as much of any of them mixed with water as she could be persuaded to to take. Her medicines were spt. Minder. and occasionally alkalies, and, as necessity indicated, opiates.

17th Her situation was similar to that of yesterday. On the 18th her hæmorrage ceased, and her pulse flagged exceedingly, so that she appeared to be on the borders of death. For three or four days she has lain constantly on her back, with her knees drawn; and whenever sh fell asleep, her feet slipped down over the end of the bed. To-day she sleeps with her eyes half closed, and her chin fallen. She has had two or three stools every day for some time past, naturally, or procured by injection. Soon after the hæmorrhage had wholly ceased, the black-vomit came on again, resembling very exactly coffee grounds, except that it was of a little darker colour. On the 20th she had black-vomit, which resembled soot sprinkled in mucus, and diluted with water; it was also somewhat flaky.

21st. This morning her tongue was covered with black furr, and black matter appeared around her cheeks and gums. She vomited some blood, as the nurse said, the last night or this morning, and in the evening had a return of the black-vomit. Her perspiration is of a highly cadaverous smell. She is troubled occasionally with singultus, but it is chiefly after taking drinks. She has been frequently delirious within the three or four last days. It was not a delirium of the wild ferocious kind; it resembled silliness or fatuity. She takes nothing except the tinct. opii, and brandy and water. Her respiration is very deep, slow and heavy. The interval between each respiration was about half a minute, her breast rising very much. During the day she made several attempts to get up and walk about the room.

23d. Her hæmorrhage returned; she takes no notice of any thing whatever. About this time an enlargement and induration of the right parotid gland took place, and pretty rapidly increased. Dr. White, of Hudson, who visited her in consultation with us, advised fomentations of warm brandy and vinegar from her feet to her neck. This was done, and repeated once or twice.

Her pulse is fuller, stronger, and more regular than yesterday; her tongue is furred: a feverish heat exists in the skin: she complains of great soreness in the throat, and difficulty of swallowing: she hiccoughs occasionally: her perspiration is cadaverous to a very uncommon degree: her mind is quite rational. During the following night the symptoms were more unfavourable than ever.

In

In addition to great debility, she was very incoherent in her ideas, was troubled with frequent hiccoughing, and constant uneasiness at the stomach: her throat grew more and more sore; and her tongue began to assume an aphthous appearance.

25th. This morning she appeared in all respects better, except about her tongue and throat: her pulse is quite regular, is not very frequent, and has considerable firmness: her skin is temperate: her respiration is less difficult: her countenance has some degree of animation in it: she is entirely rational: has had natural stools yesterday and to-day: she had her clothes changed without inconvenience, and relished a cup of coffee; but her tongue was more aphthous. In the evening her pulse was much feebler, and her tongue was covered with aphthæ. To relieve this complaint various gargles were used, but none answersd so effectually as strong gargles of yeast, or yeast mixed with sage tea. Between this time and the 27th, two or three distinct crops of aphthæ successively sprung up, and were subdued. Her throat became extremely troublesome. She had a constant drivelling from the mouth of tough mucus, which was exceedingly nauseous and offensive. The parotid gland had become as large, or larger, than a hen's egg, and very hard: her countenance had been gradually changing from a dingy hue to a deep yellow. Her urine, for a week or more, has been of the colour of strong ley.

29th. She is very uneasy at the stomach, owing, as she supposes, to the mucus from her throat getting into her stomach during sleep. This uneasiness was considerably relieved by alkalies and epispastics. In the course of the forenoon she voided several times urine of a deep green colour. Several persons who saw it, and one of them a lady very intelligent and accurate in her observations, compared it to the colour of the leaves of the yellow pine, or to the dye which is sometimes made in this part of the country from the leaves of hickory. In the afternoon she voided some of a less green colour, part of which I saw. It's colour resembled very exactly that kind of green which the leaves of Indian corn often assume after a drought, in the months of August or September, and which the corn in this part of the country had at this time assumed. It was as thick as common mucilage of gum-arabic. Into a small quantity of it we threw a few grains of the vegetable alkali: no effervescence took place. In the course of the day she expectorated two two or three tough pieces of concreted mucus, resembling the membrane which is sometimes thrown off from the larynx in cynanche trachealis. In the evening the uneasiness of the stomach increased. Alkalies were continued, and a gentle cathartic of *pulv. rhei.* and *tart. vit.* administered, and an epispastic applied upon the abdomen.

31st. Her tongue is putting on a natural and healthy appearance: her eyes had become of a deep orange-colour, and glassy.

September 1st. She informs me that, from the time she was taken sick to within about four days, she had no recollection of any thing that had passed, excepting a single circumstance respecting her husband's death. From this time she continued to mend slowly: her appetite and strength began gradually to return. She took freely of columbo, bark, wine, &c. In a few days she got quiet sleep without opiates; 13th she rode out; and now, Oct. 10th, enjoys good health.

The enlargement and induration of the parotid gland excited considerable uneasiness and apprehension. At times it was considerably painful, particularly internally. Mercurial frictions were applied below it freely, and after some time it dispersed.

### Case II.

The second person who was attacked with this disease was Mr. H. a repectable merchant in this place. He was forty-four years of age, tall, large, and well-proportioned: he was very muscular, and possessed a vigorous habit: his pulse was naturally very full, slow and regular. For several days previous to his sickness he complained of being slightly indisposed; and his brother, who was accustomed to do business with him daily, informs me that, for a week before his seizure, his breath was extremely offensive to the smell. So much was this the case, that he repeatedly advised his taking medicine, lest he should become sick. Wednesday, August 10th, in the morning, he sailed in a sloop up to Hudson. The weather was very hot and sultry. Having considerable business to transact, he walked up and down Hudson streets till he was much fatigued. In the afternoon he spent a little time with some friends, and was unusually cheerful and animated in his conversation. He returned on board the sloop between eight and nine o'clock in the evening. It was damp and chilly on board. She lay becalmed in the river several hours.

hours. About ten o'clock he was seized with a severe chilly fit, which was soon followed by great pain in the fore part of the head and eyes, in his loins, hips, and thighs; but it was most severe in his knees, and in that part of the leg where the gastrocnemius and soleus muscles unite to form the tendo-achillis. The pain he described as being more severe than all he had suffered in his life before. Early the next morning, viz. August 11th, the vessel returned to Catskill, and he was brought home. About eight A. M. he sent to Dr. Croswell, who found him with a pulse full, slow, and hard: his tongue was covered with a pretty thick whitish furr: he complained of great pain in those parts of the body before-mentioned, but said it was less severe than last night: *his face was considerably swollen* and flushed: his eyes were of a fiery red colour, and greatly pained by light: his skin was very hot: he was extremely sick at the stomach, and complained much of a burning heat in that organ: his bowels were costive.

The Doctor administered an emetic of *tartar emetic* and *ipecac. pulv.* which operated very well, and brought away a considerable quantity of bile. After a proper interval he gave him a liberal dose of calomel, and a second dose in the afternoon: it purged him considerably, but it brought away but little bile. After its operation was over he passed scybals in considerable quantity.

Friday, August 12th. This morning calomel and castor oil were administered, and operated pretty well. I saw him in consultation, at three o'clock P. M. His pains were much less severe; *his face was much swollen,* so that his eyes were half closed. This was so very remarkable that every person who came in observed it, and concluded that all the family must have been poisoned. His skin was hot and dry: his tongue was covered with a thick whitish furr: his pulse was very irregular. The first three or four strokes that I felt were full, slow, and hard: to these succeeded a small quick pulsation, and then a limping or hobbling pulse. Upon examining it a little longer, we found that it was not regular in any respect. The hobbling stroke, however, occurred as often as in one pulsation out of five.

Dr. Rush has, in some of his works, mentioned a pulse of this kind. How he defines it I do not recollect; but in this patient, in whom I observed it frequently, it seemed as though, during the first part of the stroke, there would be a regular full pulsation, but it soon dropped away, and the the remainder of the stroke was small, and dragged feebly after. He was very uneasy and restless, was troubled with constant nausea, frequent retching, and occasionally with vomiting. He was continually tossing from one side of the bed to the other; and was considerably stupid. It appeared as if the miasmata which produced the disease had acted with so much virulence and force as to produce, in a great measure, a prostration of excitement.

The propriety of blood-letting was canvassed, but it was thought prudent to defer it. Calomel was ordered in small and frequent doses. The spt. Mind. and occasionally alkalies, were made use of to relieve the stomach sickness. His drinks were the same as in Case I. but chicken water appeared to sit easier upon the stomach than any thing else.

Saturday, 13th. He was in no respect altered for the better, except that he had little or no pain. He was very uneasy. For a minute or two he would sit up, then lie down, and toss about the bed, seeking to find some relief from a change of posture. The whites of his eyes were considerably yellow. His pulse being still too active, blood-letting was again proposed, but it was thought prudent to defer it for a few hours, to take the exacerbation. No distinct remission had taken place since the commencement of the disease, and nothing like exacerbation took place to-day, except that about twelve o'clock, the face became a little more flushed.

Dr. Croswell bled him at this time in the arm, from a large orifice, about ℥x. The blood flowed freely. Three hours after, on examining it, we found a thin dense, livid skin on it, extending over the whole surface. There was not the least separation of the serum and crassamentum. The whole mass was of one uniform appearance and consistence, soft and fragile, like dissolved blood. The skin was removed, and some hours after there was about a teaspoonful of a highly yellowish serum separated, and the mass was a little more firm. It never assumed a florid colour at all. From this may we not infer that a certain healthy state of the blood is necessary in order to imbibe oxygen, or that there is some principle in the blood capable of being destroyed by disease, which, in combination with oxygen, is necessary to produce the red colour of the blood?

His pulse, for two or three hours after bleeding, was regular, soft, and slow; in other words, it was nearly quite natural. It then became one-third more frequent, quent, and less full and tremulous. This afternoon, after taking some spt. Mind. and drinking a little beer, he vomited some dark-coloured matter, of a flaky appearance; his stupor greatly increased. Vigorous efforts were made to salivate him. Sinapisms were applied to the feet, and large epispastics to the abdomen and neck. They produced some redness in the skin, but no lymph was raised, except here and there a little of an orange colour. This afternoon entire suppression of urine came on, and no stools could be obtained either by cathartics or the most stimulating injections, though both were administered liberally.

Sunday, 14th. In the morning his stupor had increased almost to coma. A streak of a dingy hue ran up each side of his face, from his neck along the back part of the under jaw to the temple. He had no stools nor urine passed to-day: his tongue had never cleared at all, except that a piece about as big as a half-dime peeled off, and left it red and dry underneath.

Monday morning, all his symptoms were much aggravated. His throat became exceedingly sore, so that he could not swallow a tea spoonful of water without great agitation, and threatening symptoms of strangulation; his respiration was very difficult, and soon became extremely laborious. Subsultus tendinum came on early this morning, and increased till every part of his body was affected with violent convulsions, and he died in vast agitation about four P. M. His face had become of a dirty yellow colour.

### Case III.

Emma, servant in Mr. H's family, was next attacked with this disease. She was taken sick early in the morning, Agust 11th. I saw her but twice, and cannot describe her case minutely. Dr. C. informs me, that she complained of great pain in the fore part of the head, and in the eyes, loins, and hips; that her eyes were very red; that she complained much of sickness at the stomach; that her fever was dispossessed of alternate *remissions* and exacerbations: in short, that it was a clearly marked case of yellow fever, but less violent than either of the preceding cases. She was entirely recovered in ten or twelve days.

### Case. IV.

About the same time that Emma was seized, Conright Briggs, a ship-carpenter, began to complain of the symptoms of this disease. I did not see him at all during the month month of August. Dr. C. considered his as a decided case of the disease. After some time he began to convalesce, and as he recovered his health he used ardent spirits much too freely. He gained strength very slowly indeed. Thursday, the 8th of September, he was employed a considerable part of the day in hunting, in consequence of which he became much fatigued. Sunday, 11th, he walked about town most of the forenoon, making preparations for a journey. The weather was hot, and he complained of being much wearied. About half past four P. M. he was seized with fever. I saw him at this time. His pulse was considerably full, hard, and frequent. His eyes were of a dull, or, perhaps, more properly, of a *yellowish* red: his face was much flushed: his skin imparted a sensation like the incipient stinging of nettles: he complained of no pain or sickness at the stomach, but he was very restless, uneasy, and desponding, and continually tossing about the bed: he was also unusually stupid. His bowels, he said, were very free, and had been so for several days. A small quantity of tartar emetic in solution was administered, together with some warm drinks, which soon sweated him. About an hour after I called in again: he lay quiet, and said he felt much easier, and recollected that he had no stools within two or three days. Calomel was given to operate as a cathartic, and afterwards in small and frequent doses, so as, if possible, to affect the system. I saw him occasionally after this, but not regularly. In the evening an entire suppression of urine took place. During the next evening, viz. 13th, he was affected with black-vomit to a great degree. On the morning of the 14th he threw up an enormous quantity of it. At this time he was extremely stupid. About eleven o'clock A. M. he said he felt much better, and was hungry, and called for some bread and meat. Some person gave him some toast and water, of which he ate a little. He almost instantly became very uneasy. I saw him about this time. He appeared to be much distressed. His eyes were yellow: his countenance had assumed, to a remarkable degree, that tawney hue so frequent in yellow fever: his extremities were cold, and bedewed with clammy sweats: he was affected with violent subsultus tendinum, and died shortly after.

[ To be continued. ]

